# Effects of Adult Feeding Treatments on Longevity, Fecundity, Flight Ability, and Energy Metabolism Enzymes of *Grapholita molesta* Moths

**DOI:** 10.3390/insects13080725

**Published:** 2022-08-13

**Authors:** Sha Su, Xiaohe Zhang, Chengzhi Jian, Baojian Huang, Xiong Peng, Marc J. B. Vreysen, Maohua Chen

**Affiliations:** 1 Key Laboratory of Integrated Pest Management on Crops in Northwestern Loess Plateau, Ministry of Agriculture and Rural Affairs, State Key Laboratory of Crop Stress Biology for Arid Areas, Northwest A&F University, Yangling 712100, China; 2 Joint FAO/IAEA Programme, Entomology Unit, FAO/IAEA Agriculture & Biotechnology Laboratory, International Atomic Energy Agency, A-2444 Vienna, Austria

**Keywords:** *Grapholita molesta*, adult diets, longevity, fecundity, flight capacity

## Abstract

**Simple Summary:**

The effect of food supplements on performance of *Grapholita molesta* (Busck) (Lepidoptera: Tortricidae) moths produced in mass rearing programs is not well known. We investigated the effect of three feeding regimes of the adult moths (supplement with sterile water or 10% honey solution and starvation) on the longevity, 10-d fecundity, flight ability, and the activity of major energy metabolism enzymes in the flight muscles of *G. molesta*. The results showed that adult food supplement can make a difference to the rearing of *G. molesta*. Under starvation, the longevity, fecundity, and five flight-related parameters of *G. molesta* adults were significantly lower than those with access to sterile water and a honey solution. There was no significant difference in adult fecundity and the five flight-related parameters of *G. molesta* with access to sterile water or honey solution. We concluded that the supply of sterile water is a better food substitution when mass-rearing of *G. molesta* for some environment-friendly insect pest control tactics such as the sterile insect technique (SIT)

**Abstract:**

The oriental fruit moth, *Grapholita molesta* (Busck) (Lepidoptera: Tortricidae) is one of the most severe fruit tree pests in China, causing huge economic losses to fruit production. So far, there are few detailed reports on the rearing protocols of *G. molesta*. In this study, we compared the longevity, 10-d fecundity, flight ability, and the activity of major energy metabolism enzymes in the flight muscles of *G. molesta* under three feeding regimes (supplement with sterile water, supplement with 10% honey solution and starvation) of the adult moths. The results showed that the longevity, 10-d fecundity, and flight parameters (cumulative flight distance and time, maximum flight distance and duration, and the average flight speed) of adult moths when supplemented with sterile water or honey solution were significantly higher than those of moths that were starved. There were no significant differences in the 10-d fecundity, flight parameters, and the activity of major energy metabolism enzymes of flight muscles between moths that were supplemented with sterile water or 10% honey solution. The flight muscles of *G. molesta* mainly used carbohydrates as an energy source when sterile water and honey solution were supplemented, and the moth mainly used lipids as an energy source under starvation. Considering the cost and potential for diet contamination during mass-rearing, supplying sterile water is considered a cost effective option for food substitution of adult *G. molesta*.

## 1. Introduction

The oriental fruit moth *Grapholita molesta* (Busck) (Lepidoptera: Tortricidae) is one of the most economically destructive pest species of stone and pome fruits and has in the past century invaded different continents from its putative native range in Asia [1]. It is a tortricid fruit borer and its larval morphology and host damage are very similar to that of its close relative, the codling moth *Cydia pomonella* (L.) (Lepidoptera: Torticidae) [2]. The massive and indiscriminative use of toxic chemical insecticides to manage populations of *G. molesta* has caused resistance of the moth to various insecticides [3]. More environmentally-friendly and sustainable pest control tactics and strategies are critically needed for the integrated management of *G. molesta*. The sterile insect technique (SIT) is an environmentally-friendly control tactic, which has been successfully used in the control of some tortricid species including the European grape moth, *Lobesia botrana* (Denis & Schiffermuller) (Lepidoptera: Tortricidae) [4] and *C. pomonella* [5]). For the application of SIT, it is better that the sterile male can mate more times with wild female moths. The males of *G. molesta* can mate multiple times with females, whereas females seldom mated more than once [6]. SIT could be an effective method to control *G. molesta* [7].

The mass-rearing of high-quality male adults that can compete with the wild males in the target area is crucial for the successful application of SIT [8]. In a rearing facility, the insects should be fed on appropriate low-cost diets that ensure advantageous traits for the insects, such as adequate longevity and high fecundity [9,10,11]. The dominant traits of insects are significantly affected by the nutritional status of both the larval stage and the adult stage [7,12]. The larvae of most lepidopteran typically have extensive and large fat body tissue as they approach pupation. However, the adult dipteran and hymenopteran tend to have only small amounts of fat body after emergence [7,12]. Water dew or sugar from nectar and oozing fruit juices is enough for the reproductive processes and normal life activities of lepidopteran adults, but the dipteran and Hymenoptera adults need to be fed some foods [13]. For most insect species, in spite of the energy and nutrition accumulation in the larval stage, the adults require more or less food supplementation, which is normally called as “supplementary nutrition” [14]. The supplements are important for the development of the reproduction system and reproduction success of adult insects. In nature, the insect adults can consume honey, nectar, pollen, dew, blood, leaves, or fruits as a food supplement [15,16]. With the rearing of particular lepidopteran species on artificial diets in the laboratory or in a mass-rearing factory, if necessary, the adults receive sugar, honey, or sterile water to improve their fecundity and quality [17]. In addition to longevity and fecundity, the flight ability is also a key quality control parameter for insects under mass-rearing conditions [18] and is a parameter that can be affected by the nutritional status of adults [19,20,21].

Insects metabolize some fuels to supply energy during flight [22]. Glyceraldehyde-3-phosphate dehydrogenase (GAPDH) and 3-hydroxyacyl-CoA dehydrogenase (HOAD) are the main enzymes for the metabolism of energy substances (referred as energy metabolism enzymes) in the flight muscles of adult insects [23]. The ratio of GAPDH to HOAD activity is often used as an index to assess the use of energy substances in the flight muscles of insects. Carbohydrates are the main substance to supply energy if the GAPDH to HOAD ratio is higher than 1, whereas lipids are the main substance to supply energy if the GAPDH to HOAD ratio is significantly lower than 1 [24].

Food supplementation is considered to be necessary for the adult moth when rearing *G. molesta* with artificial diets [25]. So far, knowledge about the effect of food supplementation on moth quality of *G. molesta* is still scarce. In this study, the performance of *G. molesta* under three feeding treatments (10% honey water, sterile water, starvation) was studied. The main types of energy utilization in the flight muscle of *G. molesta* were also investigated. This study provides knowledge for the feeding treatment of adults in the artificial rearing of *G. molesta*, which is important to improve its mass-rearing.

## 2. Materials and Methods

### 2.1. Insects

The larvae of *G. molesta* were collected from peach orchards in Yangling (34°15′53′ N, 108°3′42′ E), Shaanxi Province, China from April to August 2017. The collected larvae were reared on an artificial diet (Table S1) [26] in glass tubes (Renyuan, Cangzhou, Hebei, China) with a cotton stopper to establish a laboratory colony [7]. Around 20 larvae were reared in each tube till pupation. The pupae were separated from the diet and transferred to a transparent volumetric flask (5 L) (Renyuan Company, Cangzhou, Hebei, China). During the laboratory colony rearing, 20 pairs of newly emerged adults were transferred to a transparent glass volumetric flask (5 L) for mating and egg-laying. A piece of absorbent cotton (2.5 cm × 2.5 cm) (Hualu Company, Caoxian, China) soaked with 3 mL of honey solution (10%) in a small plastic Petri dish (5 cm in diameter, 1 cm in heigh) were put at the bottom of flash to provide food supplement for the adults. There were two pieces of wax paper (Tiancheng Company, Yangling, China) (20 cm × 6 cm) that were provided in the bottom of the flash as an oviposition substrate. All the insects (larvae, pupae, and adults) were reared in a climate-controlled incubator (GZC-500A model, Youke Company, Hefei, China) at a temperature of 26 ± 1 °C, 70 ± 10% R.H. and a photoperiod of 15:9 (L:D) h. The population had been reared for 30 generations in the incubator prior to this experiment.

### 2.2. Feeding Regimes, Adult Longevity, and Fecundity

There were two different supplements that were provided to adult moths to study the effect on adult longevity and fecundity, i.e., high temperature-sterilized ultrapure water (i.e., sterile water) and 10% honey solution. The moths under starvation served as the control group. Around 120 three-days old pupae from the aforementioned laboratory colony were transferred individually into a plastic cup (7.3 and 5.2 cm in diameter at both ends, 8.5 cm in height) that was covered with cotton gauze and the pupae gender were identified [27].

For the longevity experiments, a piece (2.5 cm × 2.5 cm) (Hualu Company, Caoxian, China) of absorbent cotton that as soaked with 3 mL of honey solution (10%) or 3 mL of sterile water was placed in a plastic Petri dish (3 cm in diameter, 1 cm in height) and put at the bottom of a transparent plastic cup (240 mL, 7.3 cm, and 5.2 cm in diameter at both ends, 8.5 cm in height) that was covered with cotton gauze immediately after the emergence of the adults. To determine longevity, the survival of the adult moths in the cup was recorded every day until their death, the date of which was recorded. The absorbent cotton that was soaked with honey or sterile water was replaced every day until adult death.

For the fecundity experiments, one newly emerged unmated female and one newly emerged male were randomly paired in a transparent plastic cup (240 mL, 7.3, and 5.2 cm in diameter at both ends, 8.5 cm in height) that was covered with cotton gauze for mating and oviposition. The inner wall of the cup was covered with wax paper for egg laying. Each treatment was replicated 30 times. From previous reports, the *G. molesta* female laid around 87% eggs in the first 10 days after emergence [28], which can be used to represent the fecundity of the moth [29]. Here, we recorded the number of eggs that were oviposited at the first 10 days. The wax paper with eggs was kept in a climate-controlled incubator (GZC-500A model, Youke Company, Hefei, China) at a temperature of 26 ± 1 °C, 70 ± 10% R.H. and a photoperiod of 15:9 (L:D), and the eggs which could develop to the black head stage [30] were counted and considered as fertile eggs. We used the number of fertile eggs to represent the 10-d fecundity of the moth. The cup was replaced by a fresh one and the moths were moved to the new cup every day [7].

### 2.3. Feeding Regimes and Adult Flight Ability

Following the method that was described by Su et al. [7], the flight ability of adult moths was assessed using a 24-way computer-linked flight mill information system (Jiaduo Industry and Trade Co., Ltd., Hebi, China) under constant temperature and humidity conditions (26 °C and 70% R.H.). The flight ability of 30 four-day old male and 30 four-day old female adults from each of the two treatments (10% honey solution, sterile water) and the control group was tested. The flight parameters (maximum flight distance, cumulative flight distance, maximum flight duration, average flight speed) of all the moths were continuously monitored by the computer program (Jiaduo Industry and Trade Co., Ltd., Hebi, China) during the tests, and recorded for 12 h in the dark by the computer-linked flight mill information system.

### 2.4. Feeding Regimes and Activity of GAPDH and HOAD Enzymes

Most of the flight muscles of the adult moths are located in the thorax. The thorax of 12 male and 12 female adults from the two treatments and the control group were used to test the activity of the GAPDH and HOAD enzymes in the flight muscles [31]. The thoraxes were put in a 1.5 mL centrifuge tube with pre-cooled 0.9% normal saline. The tube was put on ice and the thoraces were ground by glass rod at a ratio of 1 g tissue to 9 mL of normal saline [32]. The homogenate in the tube was centrifuged (5804R model, Eppendorf AG, Hamburg, Germany) at 2300× *g* at 4 °C for 15 min, and the supernatant was collected by pipettor for the enzyme activity assay. The experiment was replicated three times. The activity of the GAPDH and HOAD enzymes was determined using commercial ELISA (Enzyme Linked Immunosorben Assay) kits (Jiangsu Meibiao Biotechnology Co., Ltd., Yancheng, China) and instructions of the manufacturer were followed rigorously. The absorbance value at 450 nm was measured using the microplate reader (Tecan Infinite M200, Männedorf, Switzerland). The enzyme activity of the moth sample was calculated using the standard curve that was made by the respective standards of GADPH and HOAD according to the instructions of the ELISA kits.

### 2.5. Statistical Analysis

Statistical analyses were performed using the SPSS software (IBM SPSS Statistics for Windows, version 28.0. (IBM Corp., Armonk, NY, USA). One-way analysis of variance (ANOVA) followed by Tukey’s honestly significant difference (HSD) tests (*p* < 0.05) was used to analyze the data (longevity, 10-d fecundity, and the enzyme activity) that met the assumptions of normality and homoscedasticity. A modified nonparametric Kruskal–Wallis test for zero-inflated data in R package [33] was used to analyze the data (the maximum flight distance, the cumulative flight distance, the maximum flight duration, and the average flight speed) which did not meet the assumptions of normality and homoscedasticity.

## 3. Results

### 3.1. Adult Longevity

**Figure 1:**
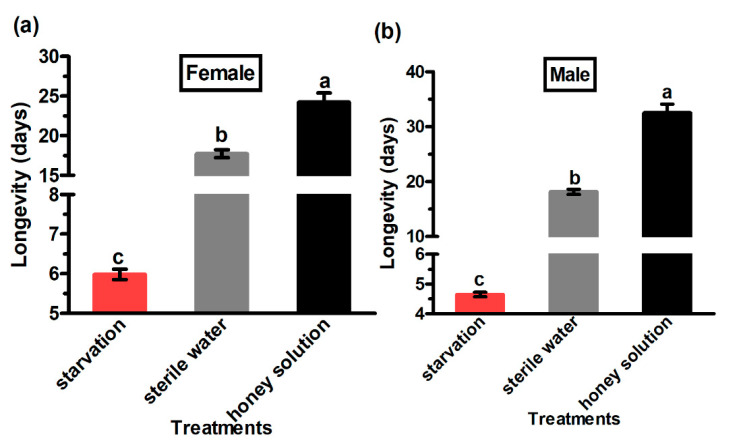
Longevity of female (**a**) and male (**b**) *Grapholita molesta* adults under different feeding treatments. The data are shown as the mean ± standard error. The different letters above the error bars indicate significant differences among the treatments (ANOVA: HSD test, *p* < 0.05).

The longevity of adult *G. molesta* males and females of the different treatments and the control group was significantly different (females—F_2,178_ = 168.42; *p* < 0.01 and males—F_2,175_ = 258.52; *p* < 0.01) (Figure 1). The moths that were provided with the honey solution had the longest longevity (24.24 days for female and 32.56 days for males), whereas the moths under starvation showed the lowest longevity (5.98 days for female and 4.65 days for males). The female and male adults that received the honey solution as supplement survived longer than the moths that received the sterile water and the control.

### 3.2. 10-d Fecundity

**Figure 2:**
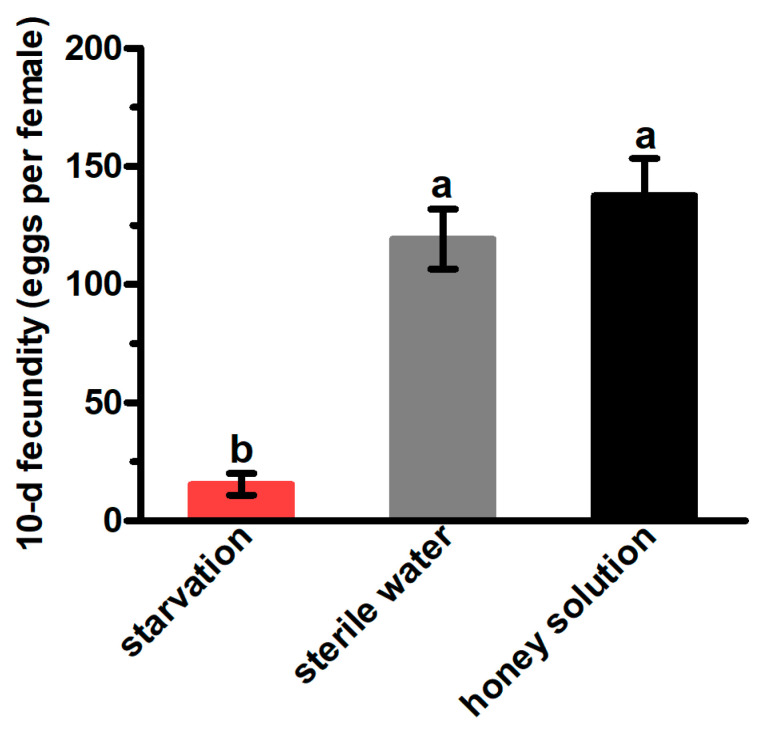
The 10-d fecundity of mated *Grapholita molesta* females under different feeding treatments. The data are shown as the mean ± standard error. The different letters above the error bars indicate significant differences between the treatments (ANOVA: HSD test, *p* < 0.05).

The 10-d fecundity (15.48 for females under starvation, 119.23 for females that received sterile water, and 137.50 for females that received 10% honey solution) of *G. molesta* females is shown in Figure 2. The 10-d fecundity under starvation was significantly lower than those that had access to the honey solution or sterile water (F_2,26_ = 27.06, *p* < 0.01), and there is no significance between the 10-d fecundity of females who had access to the honey solution and sterile water.

### 3.3. Flight Ability

**Figure 3:**
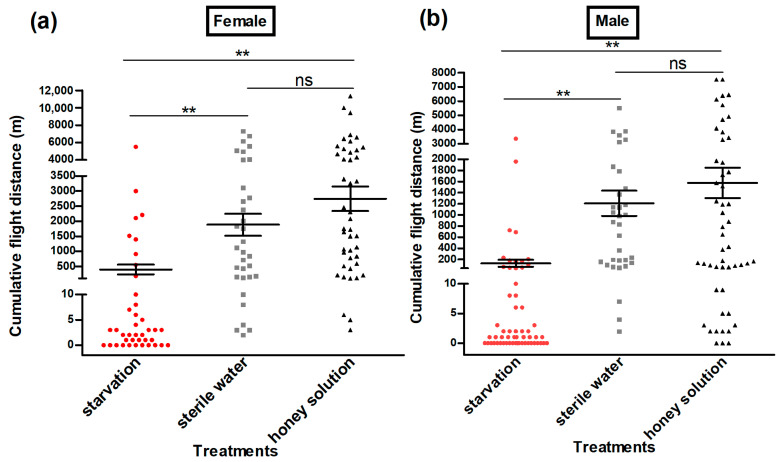
Cumulative flight distance (m) of female (**a**) and male (**b**) *Grapholita molesta* moths under different feeding treatments. The data are shown as the mean ± standard error. **, significant difference at *p* < 0.01; ns, means no significant difference.

**Figure 4:**
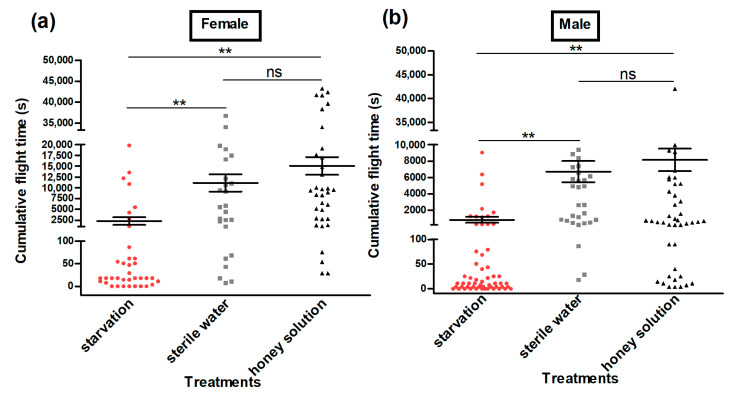
Cumulative flight time (s) of female (**a**) and male (**b**) *Grapholita molesta* moths under different feeding treatments. The data are shown as the mean ± standard error. **, significant difference at *p* < 0.01; ns, no significant difference.

**Figure 5:**
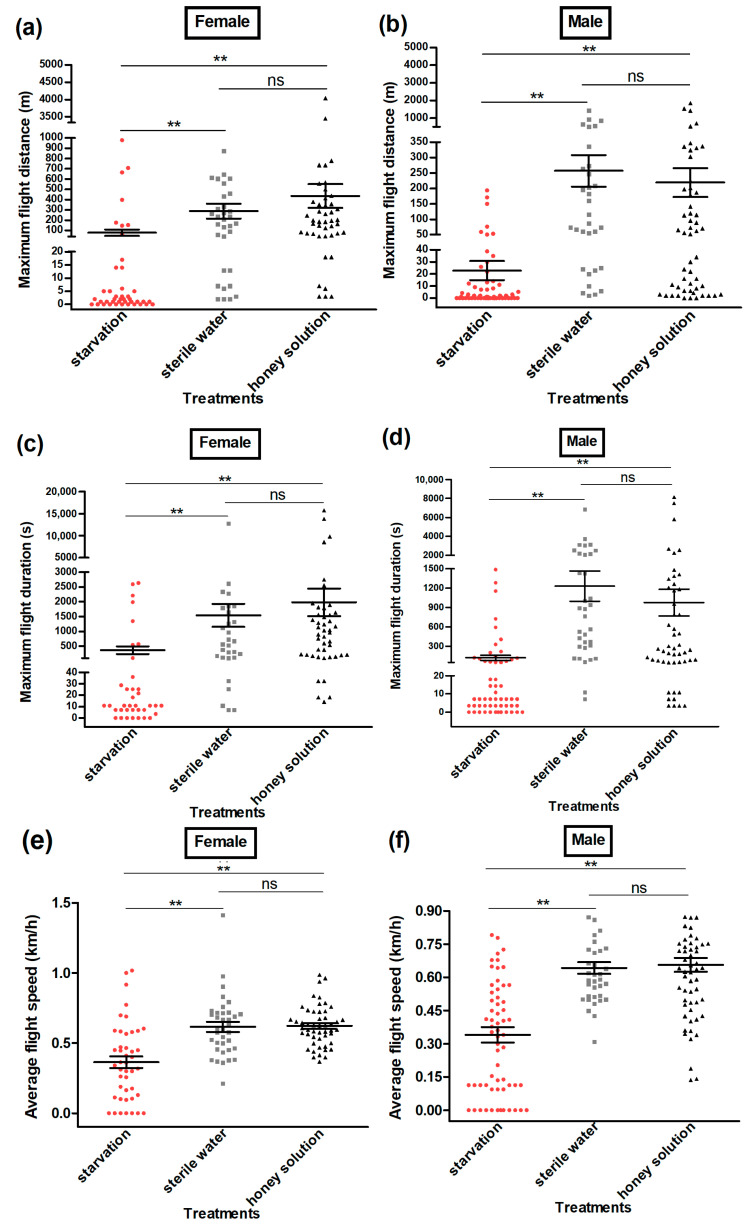
The maximum flight distance (m) (**a**,**b**), maximum flight duration (s) (**c**,**d**), and average flight speed (km/h) (**e**,**f**) of female and male *Grapholita molesta* moths under different feeding treatments. The data are shown as the mean ± standard error. **, significant difference at *p* < 0.01; ns, no significant difference.

The flight ability test of adult moths using a flight mill indicated that the cumulative flight distance (Female: Kruskal–Wallis H-test, *H* = 51.6, *p* < 0.01 (honey solution) and Kruskal–Wallis H-test, *H* = 41.6, *p* < 0.01 (sterile water); male: Kruskal–Wallis H-test, *H* = 62.6, *p* < 0.01 (honey solution) and Kruskal–Wallis H-test, *H* = 55.4, *p* < 0.01 (sterile water)) (Figure 3); the cumulative flight time (Female: Kruskal–Wallis H-test, *H* = 50.6, *p* < 0.01 (honey solution) and Kruskal–Wallis H-test, *H* = 39.8, *p* < 0.01 (sterile water); male: Kruskal–Wallis H-test, *H* = 61.6, *p* < 0.01 (honey solution) and Kruskal–Wallis H-test, *H* = 54.1, *p* < 0.01 (sterile water)) (Figure 4); the maximum flight distance (Female: Kruskal–Wallis H-test, *H* = 48.0, *p* < 0.01 (honey solution) and Kruskal–Wallis H-test, *H* = 42.6, *p* < 0.01 (sterile water); male: Kruskal–Wallis H-test, *H* = 66.5, *p* < 0.01 (honey solution) and Kruskal–Wallis H-test, *H* = 51.9, *p* < 0.01 (sterile water)) (Figure 5a,b;) the maximum flight duration (Female: Kruskal–Wallis H-test, *H* = 45.4, *p* < 0.01 (honey solution) and Kruskal–Wallis H-test, *H* = 40.6, *p* < 0.01 (sterile water); male: Kruskal–Wallis H-test, *H* = 65.6, *p* < 0.01 (honey solution) and Kruskal–Wallis H-test, *H* = 49.5, *p* < 0.01 (sterile water)) (Figure 5c,d); and the average flight speed (Female: Kruskal–Wallis H-test, *H* = 39.6, *p* < 0.01 (honey solution) and Kruskal–Wallis H-test, *H* = 36.1, *p* < 0.01 (sterile water); male: Kruskal–Wallis H-test, *H* = 50.5, *p* < 0.01 (honey solution) and Kruskal–Wallis H-test, *H* = 49.8, *p* < 0.01 (sterile water)) (Figure 5e,f) was significantly higher for the moths that had access to the honey solution and sterile water as compared with the moths that were under starvation. All the flight parameters of the female and male moths showed no significant difference between the honey solution and sterile water treatments.

### 3.4. GAPDH and HOAD Activity

**Figure 6:**
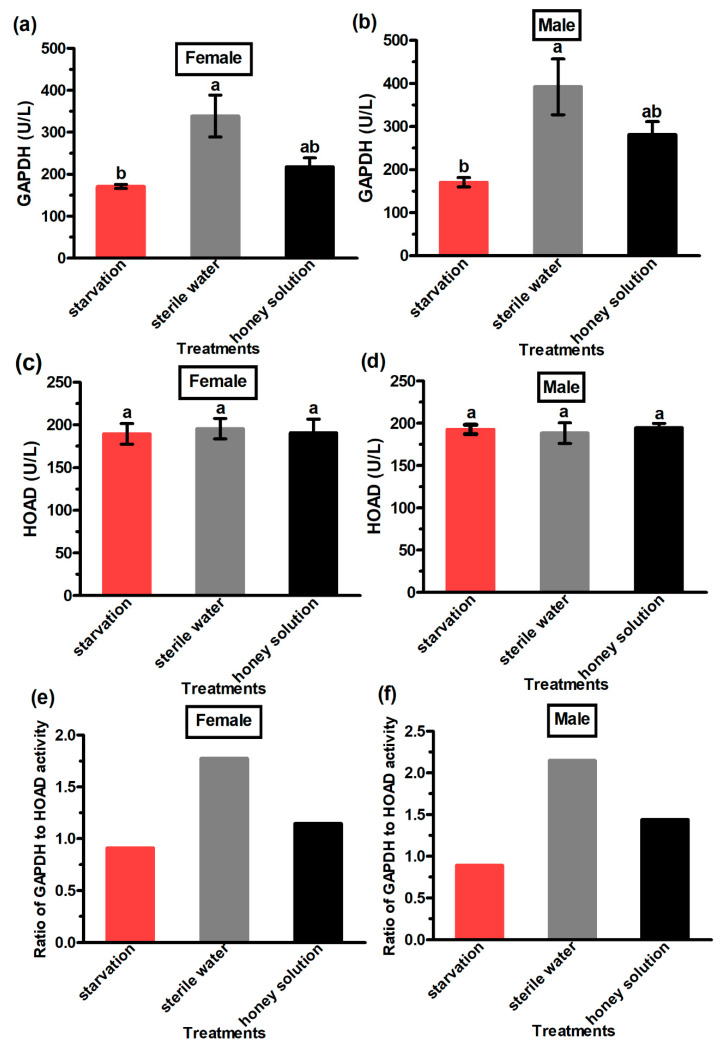
The activity (U/L) of GAPDH (**a**,**b**) and HOAD enzymes (**c**,**d**) and the ratio of GAPDH and HOAD activity (**e**,**f**) of *Grapholita molesta* female and male moths under different feeding treatments. The standard error is represented by the error bars, and different letters denote significant differences among the treatments (ANOVA: HSD test, *p* < 0.05) in graphs (**a**–**d**).

The activity of the GAPDH enzyme in the male and female adults from the sterile water treatment was significantly higher than in the starvation group (Figure 6a,b). However, the activity of the HOAD enzyme in female moths (F_2,6_ = 0.06, *p* = 0.94) and male moths (F_2,6_ = 7.06, *p* = 0.86) was not significantly different for the two treatments and the control group (Figure 6c,d). The ratio of GAPDH to HOAD activity in male and female adults was the highest for the sterile water treatment group and the lowest for the starvation group (Figure 6e,f). Moreover, the ratio of GAPDH to HOAD activity in the male and female adults was greater than 1 for the honey solution and the sterile water treatments, but lower than 1 for the starvation group.

## 4. Discussion

In this study, the longevity, fecundity, and flight ability of *G. molesta* under three feeding treatments (10% honey water, sterile water, starvation) was investigated. *G. molesta* male and female adults of the starvation group had significantly lower longevity and 10-d fecundity than those with access to the honey solution and sterile water. There was no significant difference between the fecundity and longevity of the adult moths that received honey and sterile water.

We investigated the survival of unmated moths individually, and the results showed that *G. molesta* male and female adults of the starvation group lived significantly shorter than those with access to the honey solution and sterile water. The brownheaded leafroller, *Ctenopseustis obliquana* (Walker) (Lepidoptera: Tortricidae) lived significantly longer when provided with water or honey solution in comparison with being starved [32]. Similar observations were made with some Hymenoptera for adults that received food supplements [34,35,36]. Starvation reduced the longevity of some adult Lepidoptera significantly [37,38]. These findings suggest that additional supplement for adults is important to optimize the longevity of *G. molesta*.

Food supplements significantly affected the fecundity of *C. obliquana* adults that were starved, which was lower compared with those that were fed with sterile water or a honey solution [32], as was found in this study with *G. molesta*. The fecundity of *C. obliquana* and *L. botrana* moths was not significantly different when they were supplied with sterile water and honey solution [32,37]. These data are similar to the results with *G. molesta* in the current study, i.e., starvation reduced the 10-d fecundity, and providing honey did not significantly improve the fecundity as compared with access to water. Water proved to be important for the maturation of the ovaries in female *L. botrana* and *Spodoptera exempta* (Walker) (Lepidoptera: Noctuidae) [37,38]. We also found that the eggs that were laid per day were significantly different between the honey solution treatment and the sterile water treatment except for the eggs that were laid on the third day (data not shown). Insect adults store lipids and carbohydrates as energy resources in the fat body. Newly emerged lepidopteran adults normally have a large amount of fat body. Water dew or sugar from nectar and oozing fruit juices is enough for the reproductive processes and normal life activities of lepidopteran adults [13]. Although some lepidopteran species have enough energy resources in the adult fat body, water is still important for metabolizing the lipids or carbohydrates [13]. We deduce that without enough water to support the energy metabolism, *G*. *molesta* moths under starvation will not obtain enough energy for reproductive processes and other life activities, thus resulting significant lower 10-d fecundity and shorter longevity. Although *G. molesta* females lived longer when they were provided with the honey solution, most eggs (87%) were oviposited during the first 10 days after emergence [28]. Considering the cost-effectiveness, sterile water in the mass-rearing setting seems sufficient to ensure adequate fecundity of *G. molesta* female moths when mass-rearing the moth for SIT.

The flight ability of sterile male insects is a key quality control parameter when mass-rearing moths in a factory [39]. For application the SIT against moth species, we require that the sterile male can fly some distance to locate and mate the wild female moths [39]. The *G. molesta* moth can fly several hundred meters, and a proportion of the population has the capacity to fly over 1 km and is capable of inter-habitat movement [40]. In the present study, we found that the flight ability of *G. molesta* females and males under starvation was significantly impaired as compared with those that were provided with sterile water or the honey solution. There was no significant difference in the flight ability of both sexes of moths that were provided with sterile water or the honey solution. Obviously, starvation reduced the flight ability of *G. molesta* adults considerably. Other studies came to the same conclusion, i.e., the flight ability of the olive fruit fly, *Bactrocera oleae* (Rossi) (Diptera: Tephritidae) was significantly reduced when starved [41], and newly emerged Colorado potato beetle, *Leptinotarsa decemlineata* (Say) (Coleoptera: Chrysomelidae), did not fly if they had no access to food supplements [42].

To better understand the use of energy sources during flight, we analyzed the activity of the GAPDH and HOAD enzymes in the flight muscles of *G. molesta* when they were provided with different supplements. GAPDH and HOAD are key enzymes in the glucose and lipid metabolism of insects, respectively. For instance, the GAPDH to HOAD activity ratio in the flight muscles of *Spodoptera exigua* (Hubner) (Lepidoptera, Noctuidae) and *Calliphora erythrocephala* Meigen (Diptera: Calliphoridae) was greater than 1, indicating that carbohydrates are the main energy source during flight of these two species [24,43]. In our study, we found that the GAPDH to HOAD activity ratio was greater than 1 when they were provided with sterile water or the honey solution. This suggests that the possible energy source of *G. molesta* during flight was mainly sugars from the honey solution. However, the GAPDH to HOAD activity ratio was less than 1 under starvation, indicating that lipids seem to be the main energy source during the flight of *G. molesta* under starvation.

## 5. Conclusions

Overall, our study shows that adult water or honey supplement can make a difference to the mass-rearing of *G. molesta*, i.e., the additional water and honey significantly increased the longevity, 10-d fecundity, and flight ability and influenced the major energy metabolism enzymes in the flight muscles of *G. molesta*. Under starvation, the longevity, 10-d fecundity, and five flight-related parameters of *G. molesta* adults were significantly lower than those with access to sterile water and a honey solution. The results indicate the importance of additional water or honey when mass-rearing *G. molesta* on artificial diets. There was no significant difference in the 10-d fecundity and the five flight-related parameters of *G. molesta* with access to sterile water or honey solution. As honey or sugar can cause mold contamination, considering the cost and potential contamination during mass-rearing, the supply of sterile water seems the better food substitution for the adults of *G. molesta*.

## Data Availability

The data that are presented in this study are available on request from the corresponding author.

## Supplementary Materials

The following supporting information can be downloaded at: https://www.mdpi.com/article/10.3390/insects13080725/s1, Table S1: The species studied.

## References

[1] Kirk H., Dorn S., Mazzi D. (2013). Worldwide population genetic structure of the oriental fruit moth (*Grapholita molesta*), a globally invasive pest. BMC Ecol..

[2] Zheng Y., Peng X., Liu G.M., Pan H.Y., Dorn S., Chen M.H. (2013). High genetic Diversity and structured populations of the oriental fruit moth in its range of origin. PLoS ONE.

[3] Monteiro L.B., Witt L.G., Guiloski I.C., dos Santos R.S.S., de Assis H.C.S. (2020). Evaluation of resistance management for the oriental fruit moth (Lepidoptera: Tortricidae) to insecticides in Brazilian apple orchards. J. Econ. Entomol..

[4] Simmons G.S., Sepulveda M.C.S., Barrios E.A.F., Villegas M.I., Jimenez R.E.M., Jerez A.R.G., Henderson R., Riffo H.D. (2021). Development of sterile insect technique for control of the European grapevine moth, *Lobesia botrana*, in urban areas of Chile. Insects.

[5] Marec F., Vreysen M.J.B. (2019). Advances and challenges of using the sterile insect technique for the management of pest Lepidoptera. Insects.

[6] Kong W.N., Wang Y., Jia X.T., Gao Y., Fan R.J., Li J., Ma R.Y. (2019). Emergence and mating behavior of the oriental fruit moth *Cydia molesta* (lepidoptera: Tortricidae) and its potential for reproduction. Ann. Soc. Entomol. Fra..

[7] Su S., Wang X.T., Jian C.Z., Ignatus A.D., Zhang X.H., Peng X., Chen M.H. (2021). Life-history traits and flight capacity of *Grapholita molesta* (Lepidoptera: Tortricidae) using artificial diets with varying sugar content. J. Econ. Entomol..

[8] Parker A.G., Dyck V.A., Hendrichs J., Robinson A.S. (2005). Principles and practice in area-wide integrated pest management. Mass-rearing for sterile insect release. Sterile Insect Technique.

[9] Calkins C.O., Parker A.G., Dyck V.A., Hendrichs J., Robinson A.S. (2005). Principles and practice in area-wide integrated pest management. Sterile insect quality. Sterile Insect Technique.

[10] Carpenter J.E., Blomefield T., Vreysen M.J.B. (2012). A flight cylinder bioassay as a simple, effective quality control test for *Cydia pomonella*. J. Appl. Entomol..

[11] Balestrino F., Puggioli A., Carrieri M., Bouyer J., Bellini R. (2017). Quality control methods for *Aedes albopictus* sterile male production. PLoS Neglect. Trop. Diseases.

[12] Guo J.W., Li P., Zhang J., Liu X.D., Zhai B.P., Hu G. (2019). *Cnaphalocrocis medinalis* moths decide to migrate when suffering nutrient shortage on the first day after emergence. Insects.

[13] Nation J.L. (2016). Insect Physiology and Biochemistry.

[14] Wei K., Wang X.Y., Yang Z.Q. (2016). Effects of supplementary nutrition on parasitism ability and developmental process of a gregarious parasitoid *Sclerodermus pupariae* (Hymenoptera: Bethylidae). For. Res..

[15] Heimpel G.E., Rosenheim J.A., Kattari D. (1997). Adult feeding and lifetime reproductive success in the parasitoid *Aphytis melinus*. Entomol. Exp. Appl..

[16] Harvey J.A., Cloutier J., Visser B., Ellers J., Wackers F.L., Gols R. (2012). The effect of different dietary sugars and honey on longevity and fecundity in two hyperparasitoid wasps. J. Insect Physiol..

[17] Hari N.S., Jindal J., Malhi N.S., Khosa J.K. (2008). Effect of adult nutrition and insect density on the performance of spotted stem borer, *Chilo partellus* in laboratory cultures. J. Pest Sci..

[18] Collins S.R., Taylor P.W. (2010). Flight ability procedures for mass-reared Queensland fruit flies, *Bactrocera tryoni*: An assessment of some variations. Entomol. Exp. Appl..

[19] Bargielowski I., Kaufmann C., Alphey L., Reiter P., Koella J. (2012). Flight performance and teneral energy reserves of two genetically-modified and one wild-type strain of the yellow fever mosquito *Aedes aegypti*. Vector Borne Zoonotic Dis..

[20] Ge S.S., He L.M., He W., Xu R.B., Sun X.T. (2019). Determination on moth flight capacity of *Spodoptera frugiperda*. Plant Prot..

[21] Shirai Y. (2006). Flight activity, reproduction, and adult nutrition of the beet webworm, *Spoladea recurvalis* (Lepidoptera: Pyralidae). Appl. Entomol. Zool..

[22] Beenakkers A.M.T., Van der Horst D.J., Van Marrewijk W.J.A. (1984). Insect flight muscle metabolism. Insect Biochem..

[23] Li K.B., Luo L.Z. (1999). Activities of enzymes in the flight muscle of pupal and adult oriental armyworm, *Mythimna separata* (Walker). Acta Entomol. Sinica.

[24] Beenakkers A.M.T. (1969). Carbohydrate and fat as a fuel for insect flight: A comparative study. J. Insect Physiol..

[25] He C., Meng Q.K., Hua L., Chen W. (2011). Rearing Technique of oriental fruit moth (Lepodoptera: Tortricidae). J. Shanghai Jiaotong Univ. (Agri. Sci.).

[26] Yu Q., Feng Y.T., Guo X.J., Guo G.M., Hao C., Fan R.J. (2017). Effects of humidity on the survival and fecundity of *Grapholita molesta* (Lepidoptera Tortricidae). Acta Entomol. Sinica.

[27] Cheng X., Wang J.Y., Zhang Q., Jiang R.D., Feng M.X. (2015). How to identify the female and male pupa and adult of the oriental fruit moth. Shanxi Fruits.

[28] Hughes J., Dorn S. (2002). Sexual differences in the flight performance of the oriental fruit moth. Entomol. Exp. Appl..

[29] Su S., Jian C.Z., Zhang X.H., Fang S.S., Peng X., Piñero J.C., Chen M.H. (2021). Sublethal effects of abamectin on the development, reproduction, detoxification enzyme activity, and related gene expression of the oriental fruit moth (Lepidoptera: Tortricidae). J. Econ. Entomol..

[30] Forte S.N., Ferrrro A.A., Alonso T.S. (2002). Content and composition of phosphoglycerols and neutral lipids at different developmental stages of the eggs of the codling moth, *Cydia pomonella* (Lepidoptera: Tortricidae). Arch. Insect Biochem. Physiol..

[31] Kim K.N., Sin U.C., Yun C.N., Song H.S., Huang Z.J., Huang Q.Y., Lei C.L. (2019). Influence of green light illumination on several enzymes involved in energy metabolism in the oriental armyworm, *Mythimna separata* (Lepidoptera: Noctuidae). J. Asia-Pac. Entomol..

[32] Stevens P., Froud K., Jamieson L. (2002). Effects of adult feeding on longevity and fecundity of *Ctenopseustis obliquana* (Lepidoptera: Tortricidae). N. Z. J. Crop Hort. Sci..

[33] Wang W.J., Chen E.Z., Li H.Z. (2021). ZIR: Modified Wilcoxon Rank Test and Kruskal Wallis Test for Zero-Inflated Data, ZIR (Zero-Inflated Rank Test) R Package Version 1.0.0. https://github.com/chvlyl/ZIR.

[34] Siekmann G., Tenhumberg B., Keller M.A. (2001). Feeding and survival in parasitic wasps: Sugar concentration and timing matter. OIKOS.

[35] Eijs I.E.M., Ellers J., Van Duinen G.J. (1998). Feeding strategies in drosophilid parasitoids: The impact of natural food resources on energy reserves in females. Ecol. Entomol..

[36] Snart C.J.P., Kapranas A., Williams H., Barrett D.A., Hardy I.C.W. (2018). Sustenance and performance: Nutritional reserves, longevity, and contest outcomes of fed and starved adult parasitoid wasps. Front. Ecol. Evol..

[37] Savopoulou-Soultani M., Milonas P.G., Stavridis D.G. (1998). Role of availability of food to the adult *Lobesia botrana* (Lepidoptera: Tortricidae) in its reproductive performance. J. Econ. Entomol..

[38] Gunn A., Gatehouse A.G. (2010). Effects of the availability of food and water on reproduction in the African army worm, *Spodoptera exempta*. Physiol. Entomol..

[39] Klassen W., Dyck V.A., Hendrichs J., Robinson A.S. (2005). Area-wide integrated pest management and the sterile insect technique. Sterile Insect Technique: Principles and Practice in Area-Wide Integrated Pest Management.

[40] Sciarretta A., Trematerra P. (2006). Geostatistical characterization of the spatial distribution of *Grapholita molesta* and *Anarsia lineatella* males in agricultural landscape. J. Appl. Entomol..

[41] Wang X.G., Johnson M.W., Daane K.N., Opp S. (2009). Combined effects of heat stress and food supply on flight performance of olive fruit fly (Diptera: Tephritidae). Ann. Entomol. Soc. Am..

[42] Weber D.C., Ferro D.N. (1996). Flight and fecundity of Colorado potato beetles (Coleoptera: Chrysomelidae) fed on different diets. Ann. Entomol. Soc. Am..

[43] Han L.Z., Zhai B.P., Zhang X.X., Liu P.L. (2005). Activity of enzymes related to energy metabolism in the flight muscle of beet armyworm. Acta Ecol. Sinica.

