# Supplementary material for: Effects of Adult Feeding Treatments on Longevity, Fecundity, Flight Ability, and Energy Metabolism Enzymes of Grapholita molesta Moths
Source: Insects. 2022 Aug 13;13(8):725. doi: 10.3390/insects13080725 (PMC9409247; doi:10.3390/insects13080725)
Supplement: Supplementary file 1 [file insects-13-00725-s001.zip › insects-1805186-supplementary.pdf]

Supplementary Materials

Table S1. Artificial diet formula that was used in this study.

Ingredient	Amounts (g)
Maize powder	75
Soybean powder	75
Yeast powder	30
Tomato ketchup	198
L-ascorbic acid	3
Cholesterol	0.1
Sorbic acid	1
Nipagin ester	2
Agar	14
Water	700
Sucrose	15
